# Supplementary material for: Potential Diagnostic and Monitoring Biomarkers of Obstructive Sleep Apnea–Umbrella Review of Meta-Analyses
Source: J Clin Med. 2022 Dec 21;12(1):60. doi: 10.3390/jcm12010060 (PMC9821668; doi:10.3390/jcm12010060)
Supplement: Supplementary file 1 [file jcm-12-00060-s001.zip › Supplementary Material Table S2.pdf]

Table S2. Summary of meta-analyses included with quality assessment.

Author	Year	Chosen serum or plasma biomarkers	Quality assessment of study - AMSTAR 2															
			(1) Question and inclusion	(2) Protocol	(3) Study design	(4) Comprehensive search	(5) Study selection	(6) Data extraction	(7) Excluded studies justification	(8) Included studies details	(9) Risk of Bias	(10) Funding sources	(11) Statistical Methods	(12) Risk of Bias in Meta-analysis	(13) Risk of Bias in Individual Studies	(14) Explanation of hetero-geneity	(15) Publication Bias	(16) Conflict of Interest
Comparison to the control group																		
Nadeem et al.[31]	2013	CRP, TNF-α, IL-6, IL-8	YES	NO	NO	PART. YES	YES	YES	NO	PART. YES	NO	NO	NO	YES	YES	YES	YES	YES
Li et al.[32]	2013	Hcy	YES	NO	NO	PART. YES	YES	YES	NO	PART. YES	PART. YES	NO	NO	YES	NO	YES	YES	YES
Niu et al.[33]	2014	Hcy	YES	NO	NO	PART. YES	YES	YES	NO	PART. YES	NO	NO	NO	YES	YES	YES	YES	YES
Nadeem et al.[34]	2014	TC, LDLc, HDLc, TG	YES	PART. YES	NO	PART. YES	YES	YES	NO	NO	NO	NO	NO	NO	NO	NO	YES	YES
De Luca Canto et al.[35]	2015	IL-6 TNF-α, IL-10	YES	YES	NO	PART. YES	YES	YES	YES	PART. YES	PART. YES	NO	YES	YES	YES	YES	YES	YES
Wang et al.[8]	2015	IL-6	YES	NO	NO	PART. YES	YES	YES	NO	PART. YES	PART. YES	NO	YES	YES	YES	YES	YES	YES
Li et al.[36]	2017	CRP	YES	NO	NO	PART. YES	YES	YES	NO	YES	NO	NO	NO	YES	YES	YES	YES	YES

Author	Year	Chosen serum or plasma biomarkers	Quality assessment of study - AMSTAR 2															
			(1) Question and inclusion	(2) Protocol	(3) Study design	(4) Comprehensive search	(5) Study selection	(6) Data extraction	(7) Excluded studies justification	(8) Included studies details	(9) Risk of Bias	(10) Funding sources	(11) Statistical Methods	(12) Risk of Bias in Meta-analysis	(13) Risk of Bias in Individual Studies	(14) Explanation of hetero-geneity	(15) Publication Bias	(16) Conflict of Interest
Li et al.[37]	2017	TNF	YES	NO	NO	PART. YES	YES	YES	NO	YES	NO	NO	YES	NO	YES	NO	YES	YES
Zhang et al.[38]	2017	VEGF	YES	NO	NO	NO	YES	NO	NO	YES	NO	NO	NO	NO	YES	YES	YES	NO
Sookoian et al[39]	2017	ALT, AST	YES	NO	NO	PART. YES	YES	NO	NO	PART. YES	NO	NO	NO	YES	YES	YES	YES	YES
Van der Touw et al.[40]	2019	CRP	YES	NO	NO	PART. YES	YES	YES	NO	PART. YES	NO	NO	NO	NO	YES	YES	YES	YES
Lu et al.[128]	2019	adiponectin	YES	YES	NO	PART. YES	YES	YES	NO	YES	YES	NO	YES	YES	YES	YES	YES	YES
Qiu et al.[41]	2020	VEGF	YES	NO	NO	PART. YES	YES	YES	NO	YES	YES	NO	YES	YES	YES	YES	NO	YES
Imani et al.[42]	2020	IL-6	YES	NO	NO	PART. YES	YES	YES	NO	YES	YES	YES	NO	YES	YES	YES	YES	YES
Imani et al.[43]	2020	TNF-α	YES	PART. YES	NO	PART. YES	YES	YES	NO	YES	YES	NO	YES	NO	YES	YES	YES	NO
Cao et al.[44]	2020	TNF-α	YES	YES	NO	PART. YES	YES	YES	NO	YES	PART. YES	NO	YES	YES	YES	YES	YES	YES

Author	Year	Chosen serum or plasma biomarkers	Quality assessment of study - AMSTAR 2															
			(1) Question and inclusion	(2) Protocol	(3) Study design	(4) Comprehensive search	(5) Study selection	(6) Data extraction	(7) Excluded studies justification	(8) Included studies details	(9) Risk of Bias	(10) Funding sources	(11) Statistical Methods	(12) Risk of Bias in Meta-analysis	(13) Risk of Bias in Individual Studies	(14) Explanation of hetero-geneity	(15) Publication Bias	(16) Conflict of Interest
Fadaei et al.[45]	2020	MDA	YES	NO	NO	PART. YES	YES	NO	NO	PART. YES	PART. YES	NO	NO	YES	YES	YES	YES	YES
Li et al.[46]	2021	IL-8	YES	YES	YES	YES	YES	YES	NO	YES	YES	NO	YES	YES	YES	YES	YES	YES
Imani et al.[47]	2021	CRP	YES	NO	NO	NO	YES	YES	NO	PART. YES	PART. YES	NO	YES	YES	YES	YES	YES	YES
Li et al.[48]	2021	Leptin	YES	YES	NO	PART. YES	YES	YES	NO	YES	PART. YES	NO	YES	YES	YES	YES	YES	YES
Imani et al.[132]	2021	Cortisol	YES	PART. YES	NO	PART. YES	YES	YES	NO	YES	PART. YES	NO	YES	YES	YES	YES	YES	YES
Yi et al.[49]	2022	IL-8	YES	NO	NO	PART. YES	YES	YES	NO	YES	YES	NO	YES	YES	YES	YES	YES	YES
Yi et al.[50]	2022	CRP, TNF-α	YES	NO	NO	PART. YES	YES	YES	NO	PART. YES	PART. YES	NO	YES	YES	YES	YES	YES	YES
He et al.[51]	2022	IGF-1	YES	NO	NO	PART. YES	YES	YES	NO	PART. YES	PART. YES	NO	YES	YES	YES	YES	YES	YES
Change after treatment - CPAP or sleep surgery																		
Hecht et al.[131]	2011	adiponectin	YES	PART. YES	YES	PART. YES	YES	YES	NO	PART. YES	PART. YES	NO	NO	NO	YES	NO	NO	YES

Author	Year	Chosen serum or plasma biomarkers	Quality assessment of study - AMSTAR 2															
			(1) Question and inclusion	(2) Protocol	(3) Study design	(4) Comprehensive search	(5) Study selection	(6) Data extraction	(7) Excluded studies justification	(8) Included studies details	(9) Risk of Bias	(10) Funding sources	(11) Statistical Methods	(12) Risk of Bias in Meta-analysis	(13) Risk of Bias in Individual Studies	(14) Explanation of hetero-geneity	(15) Publication Bias	(16) Conflict of Interest
Friedman et al.[52]	2012	CRP	YES	NO	YES	PART. YES	YES	YES	NO	YES	PART. YES	NO	YES	YES	YES	YES	YES	YES
Xie et al.[53]	2013	CRP, IL-6, IL-8, TNF-α	YES	NO	NO	PART. YES	YES	YES	NO	PART. YES	NO	NO	NO	YES	YES	YES	YES	YES
Guo et al.[54]	2013	CRP	YES	PART. YES	NO	YES	YES	YES	NO	PART. YES	NO	NO	NO	NO	YES	YES	YES	YES
Baessler et al.[55]	2013	CRP, TNF-α, IL-6	YES	PART. YES	YES	PART. YES	YES	YES	NO	PART. YES	NO	NO	NO	NO	NO	NO	NO	YES
Xu et al.[56]	2014	TC, LDLc, HDLc, TG	YES	PART. YES	YES	PART. YES	YES	YES	NO	YES	YES	NO	YES	YES	YES	YES	YES	YES
Chen et al.[57]	2014	HCY	YES	NO	NO	PART. YES	YES	YES	NO	YES	NO	NO	YES	YES	NO	YES	YES	YES
Chen et al.[58]	2014	IGF-1	YES	NO	NO	PART. YES	YES	YES	NO	PART. YES	NO	NO	NO	NO	NO	YES	YES	YES
Zhang et al.[59]	2014	Leptin	YES	NO	NO	PART. YES	YES	YES	NO	YES	NO	NO	NO	NO	YES	YES	YES	YES
Chen et al.[60]	2015	Leptin	YES	NO	NO	PART. YES	NO	NO	NO	PART. YES	NO	NO	NO	NO	NO	YES	YES	YES

Author	Year	Chosen serum or plasma biomarkers	Quality assessment of study - AMSTAR 2															
			(1) Question and inclusion	(2) Protocol	(3) Study design	(4) Comprehensive search	(5) Study selection	(6) Data extraction	(7) Excluded studies justification	(8) Included studies details	(9) Risk of Bias	(10) Funding sources	(11) Statistical Methods	(12) Risk of Bias in Meta-analysis	(13) Risk of Bias in Individual Studies	(14) Explanation of hetero-geneity	(15) Publication Bias	(16) Conflict of Interest
Iftikhar et al.[130]	2015	adiponectin	YES	YES	YES	NO	NO	YES	NO	PART. YES	PART. YES	NO	YES	YES	NO	YES	YES	YES
Chen et al.[129]	2015	adiponectin	YES	PART. YES	NO	PART. YES	YES	YES	NO	YES	PART. YES	NO	YES	YES	NO	YES	YES	YES
Zhong et al.[61]	2016	IL-6	YES	PART. YES	NO	YES	YES	YES	NO	PART. YES	NO	NO	NO	NO	NO	YES	YES	YES
Chen et al.[62]	2018	ALT AST	YES	PART. YES	NO	PART. YES	YES	YES	NO	YES	NO	NO	NO	NO	NO	YES	YES	YES
Qi et al.[63]	2018	VEGF	YES	YES	YES	PART. YES	YES	YES	NO	PART. YES	YES	NO	NO	YES	YES	YES	YES	YES
Ning et al.[64]	2019	hsCRP, IL-6, TNF-α	YES	PART. YES	YES	YES	YES	YES	NO	PART. YES	NO	NO	YES	YES	NO	YES	YES	YES
Chen et al.[65]	2020	MDA	YES	PART. YES	NO	YES	YES	YES	NO	YES	NO	NO	NO	NO	NO	YES	YES	YES
Fadaei et al.[66]	2020	MDA	YES	YES	NO	YES	YES	YES	NO	YES	NO	NO	YES	YES	YES	YES	YES	YES
Kang et al.[67]	2021	CRP	YES	YES	NO	PART. YES	YES	YES	NO	YES	YES	NO	YES	YES	YES	YES	YES	YES

Author	Year	Chosen serum or plasma biomarkers	Quality assessment of study - AMSTAR 2															
			(1) Question and inclusion	(2) Protocol	(3) Study design	(4) Comprehensive search	(5) Study selection	(6) Data extraction	(7) Excluded studies justification	(8) Included studies details	(9) Risk of Bias	(10) Funding sources	(11) Statistical Methods	(12) Risk of Bias in Meta-analysis	(13) Risk of Bias in Individual Studies	(14) Explanation of hetero-geneity	(15) Publication Bias	(16) Conflict of Interest
Lee et al.[68]	2021	TC, LDLc, HDLc, TG	YES	YES	NO	PART. YES	YES	YES	NO	YES	YES	NO	YES	YES	YES	YES	YES	YES
Tian et al.[69]	2021	ICAM-1, VCAM-1	YES	NO	NO	PART. YES	YES	YES	NO	YES	YES	NO	NO	YES	NO	YES	YES	YES
Ken-Dror et al.[133]	2021	Cortisol	YES	NO	NO	PART. YES	YES	YES	NO	PART. YES	PART. YES	NO	YES	YES	YES	YES	YES	YES
Wang et al.[70]	2022	CRP TNF-α, lipid profile	YES	YES	YES	PART. YES	YES	YES	NO	PART. YES	YES	NO	NO	YES	NO	YES	YES	YES
Lee et al.[71]	2022	TNF-α, IL-6	YES	YES	NO	PART. YES	YES	YES	NO	YES	YES	NO	NO	YES	YES	YES	YES	YES
Lee et al.[72]	2022	lipid profile	YES	YES	NO	PART. YES	YES	YES	NO	YES	YES	NO	NO	YES	YES	YES	YES	YES
Yeo et al.[73]	2022	CRP, lipid profile, TNF-α, IL-6	YES	YES	NO	YES	YES	YES	NO	YES	YES	NO	YES	YES	YES	YES	YES	YES

Author	Year	Chosen serum or plasma biomarkers	Quality assessment of study - AMSTAR 2															
			(1) Question and inclusion	(2) Protocol	(3) Study design	(4) Comprehensive search	(5) Study selection	(6) Data extraction	(7) Excluded studies justification	(8) Included studies details	(9) Risk of Bias	(10) Funding sources	(11) Statistical Methods	(12) Risk of Bias in Meta-analysis	(13) Risk of Bias in Individual Studies	(14) Explanation of hetero-geneity	(15) Publication Bias	(16) Conflict of Interest

Abbreviations: CRP, C-reactive protein; TNF-α, , Tumor Necrosis Factor alpha; IL-6, Interleukin-6; IL-8, Interleukin-8; Hcy, homocysteine; TC, total cholesterol; LDLc, low-density lipoprotein cholesterol; HDLc, high-density lipoprotein cholesterol; TG, triglyceride; IL-10, Interleukin-10; VEGF, vascular endothelial growth factor; ALT, alanine transaminase; AST, aspartate aminotransferase; MDA, malondialdehyde; IGF-1, insuline-like growth factor; hsCRP, high-sensitivity C-reactive protein;

Colour	Meaning
(green)	Yes
(orange)	No
(yellow)	Part, Yes
